# Prognostic landscape of tumor-infiltrating immune cells and immune-related genes in the tumor microenvironment of gastric cancer

**DOI:** 10.18632/aging.103519

**Published:** 2020-09-23

**Authors:** Shichao Zhang, Zhu Zeng, Yongfen Liu, Jiangtao Huang, Jinhua Long, Yun Wang, Xiaoyan Peng, Zuquan Hu, Yan Ouyang

**Affiliations:** 1 Immune Cells and Antibody Engineering Research Center of Guizhou Province, Key Laboratory of Biology and Medical Engineering, School of Biology and Engineering/School of Basic Medical Sciences, Guizhou Medical University, Guiyang 550025, Guizhou, P.R. China; 2 Key Laboratory of Environmental Pollution Monitoring and Disease Control, Ministry of Education, Guizhou Medical University, Guiyang 550025, Guizhou, P.R. China; 3 Affiliated Tumor Hospital, Guizhou Medical University, Guiyang 550004, Guizhou, P.R. China

**Keywords:** gastric cancer, tumor-infiltrating immune cells (TIICs), immune-related genes (IRGs), prognostic value, tumor microenvironment

## Abstract

The tumor microenvironment is closely related to the progression and immune escape of tumor cells. Tumor-infiltrating immune cells (TIICs) and immune-related genes (IRGs) are indispensable components of the tumor microenvironment and have been demonstrated to be highly valuable in determining the prognosis of multiple cancers. To elucidate the prognostic value of TIICs and IRGs in gastric cancer, we conducted a comprehensive analysis focusing on the abundances of 22 types of TIICs and differentially expressed IRGs based on a dataset from The Cancer Genome Atlas (TCGA). The results showed that great composition differences in TIICs and immune cell subfractions were associated with survival outcomes in different stages. Additionally, 29 hub genes were characterized from 345 differentially expressed IRGs and found to be significantly associated with survival outcomes. Then, an independent prognostic indicator based on ten IRGs was successfully constructed after multivariate adjustment for some clinical parameters. Further validation revealed that these hub IRGs could reflect the infiltration levels of immune cells. Thus, our results confirmed the clinical significance of TIICs and IRGs in gastric cancer and may establish a foundation for further exploring immune cell and gene targets for personalized treatment.

## INTRODUCTION

Gastric cancer is one of the most common human malignancies of the digestive system and ranks as the third leading cause of cancer-related death worldwide, particularly in East Asia [1, 2]. It is heterogeneous, and a recent study by The Cancer Genome Atlas (TCGA) developed a robust molecular classification system for gastric cancer [3]. Although the survival rate of early gastric cancer has continuously improved in recent years due to advancements in treatment techniques and regimens, the low rate of early diagnosis means that the best surgical window is missed in most patients [1]. For advanced-stage patients, immunotherapy is considered to be one of the most promising treatments. Unfortunately, immunotherapies based on dendritic cells (DCs), chimeric antigen receptor T cells (CAR-T cells) and immune checkpoints are not always effective due to tumor heterogeneity and the complicated tumor microenvironment (TME). Therefore, it is beneficial to study the deviations in the immune cell landscape for designing personalized treatment regimens or exploring new drug targets for gastric cancer.

Innate immunity is an important first line of defense against infectious agents and tumors and consists of the immunological barrier, immune cells, and immune molecules. Natural killer (NK) cells, macrophages, DCs, mast cells, eosinophils and neutrophils are the main innate immune cells. In cancer patients, tumor antigens can activate the body’s adaptive immune response as the primary and decisive force in the elimination of tumors. Adaptive immunity is composed of two important branches: T cell-mediated cellular immunity and antibody-mediated humoral immunity. Tumor-infiltrating immune cells (TIICs) are indispensable components of the TME and play important roles in tumorigenesis and progression. Thus, TIICs have been widely applied for the clinical prediction of cancer treatment [4–7]. Previous studies concerning alterations in the composition of immune cells in gastric cancer mainly rely on immunohistochemistry or flow cytometry [8–10], which only detect a few immune cell types at once and are limited by phenotypic markers and the number of samples. Moreover, TIICs may have diverse influences on tumor progression, invasion and metastasis in different cancer types or even in different patient subgroups. Thus, it is difficult to judge the clinical implications of TIICs based on limited detection data [11].

In recent years, large amounts of gene expression data for primary tumors from cancer patients have been collected. Newman et al. introduced CIBERSORT as an analytical method for characterizing the abundances of member cell types in a mixed cell population from their gene expression profiles [12]. Subsequently, this method has been further developed to estimate the composition of infiltrated immune cells in different types of cancer, such as breast cancer, lung cancer and renal cell carcinoma [4–6, 13, 14]. In addition, recent studies revealed that immune-related genes (IRGs) are closely related to TIICs and exhibit considerable promise in survival prediction for multiple cancers [15–19]. However, the clinical relevance and prognostic significance of the immune cell composition and IRGs in gastric cancer remain under exploration.

In this study, the aim was to estimate the clinical implications of the TIIC composition and IRGs in gastric cancer. The transcriptomic RNA-seq data were downloaded from the TCGA database [20, 21] and the immune cell composition and its prognostic value in gastric cancer were investigated. Subsequently, the expression and prognostic landscape of survival-associated IRGs were comprehensively analyzed and a prognostic signature was successfully constructed as an independent predictor for gastric cancer patients. The results of this study could provide promising insight for further exploiting biomarkers for the diagnosis and individualized treatment of gastric cancer based on TIICs and IRGs.

## RESULTS

### Differences in adaptive immune cells

**Figure 1:**
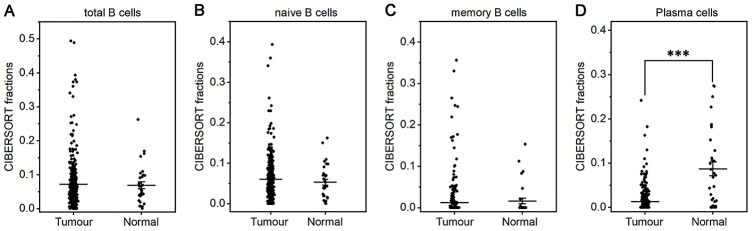
**Fractions of B cells and plasma cells in gastric cancer and normal tissue.** CIBERSORT was applied to analyze the fractions of TIICs, and each dot represents one sample. The mean±SD for each cell subtype including total B cells (**A**), naive B cells (**B**), memory B cells (**C**) and plasma cells (**D**) was calculated and compared using one-way ANOVA. ***P<0.001.

**Figure 2:**
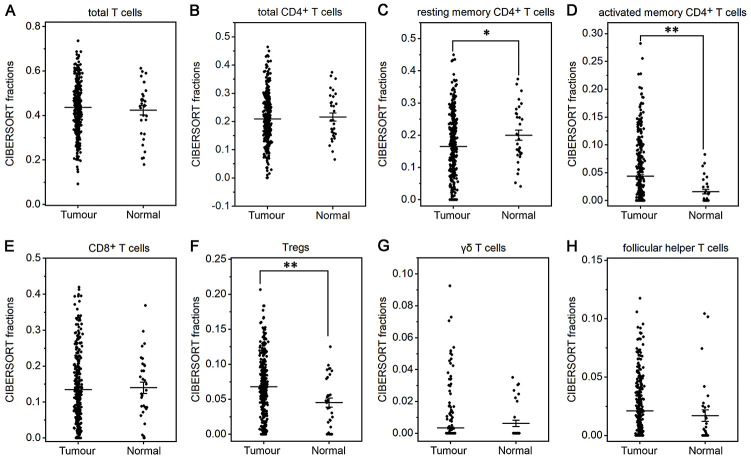
**Fractions of T cells in gastric cancer and normal tissue.** CIBERSORT was applied to determine the fractions of TIICs, and each dot represents one sample. The mean±SD for each cell subtype including total T cells (**A**), total CD4^+^ T cells (**B**), resting memory CD4^+^ T cells (**C**), activated memory CD4^+^ T cells (**D**), CD8^+^ T cells (**E**), Tregs (**F**), δγ T cells (**G**) and Tfh cells (**H**) was calculated and compared using one-way ANOVA. *P<0.05; **P<0.01.

The fraction of plasma cells was lower in gastric cancer than in normal tissue (*P*< 0.001, Figure 1D), but there were no significant differences in the fractions of total B cells, naive and memory B cells (Figure 1A–1C). These results suggest that the ability of B cells to differentiate into plasma cells is inhibited in gastric cancer, which may affect antitumor immunity. For T cell subpopulations, the fractions of activated memory CD4^+^ T cells and Tregs increased in gastric cancer compared with normal tissue (*P*<0.01, Figure 2D, 2F), while the resting memory CD4^+^ T cell fraction decreased in gastric cancer tissue (*P*<0.05, Figure 2C). However, the proportions of total T cells, CD4^+^ T cells, CD8^+^ T cells, δγ T cells and follicular helper T (Tfh) cells showed no great changes (Figure 2A, 2B, 2E, 2G, 2H). Thus, it is unlikely that a T cell-mediated antitumor immune response occurs in gastric cancer patients.

### Differences in innate immune cells

**Figure 3:**
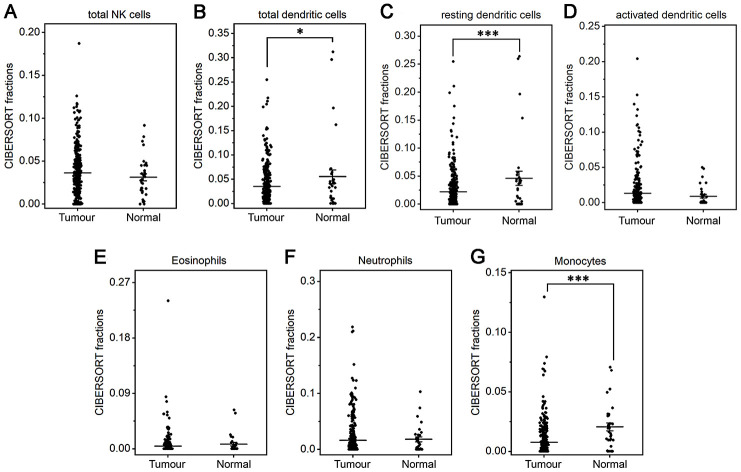
**Fractions of NK cells, DCs, eosinophils, neutrophils and monocytes in gastric cancer and normal tissue.** CIBERSORT was applied to analyze the fractions of TIICs, and each dot represents one sample. The mean±SD for each cell subtype including total NK cells (**A**), total DCs (**B**), resting DCs (**C**), activated DCs (**D**), eosinophils (**E**), neutrophils (**F**), and monocytes (**G**) was calculated and compared using one-way ANOVA. *P<0.05; ***P<0.001.

**Figure 4:**
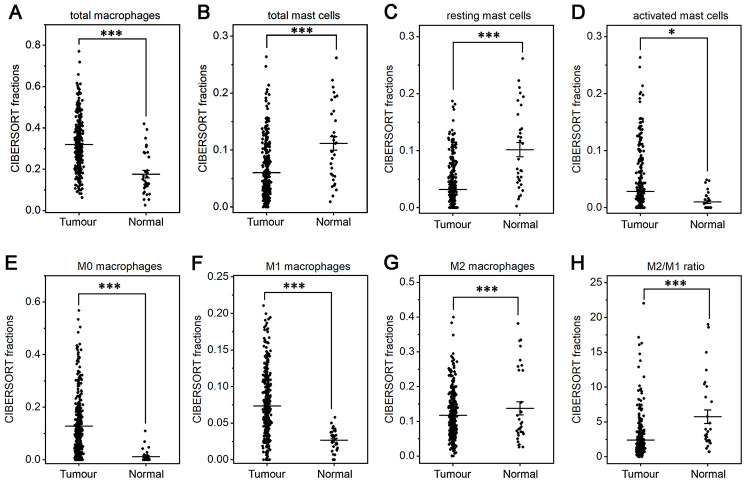
**Fractions of macrophages and mast cells in gastric cancer and normal tissue.** CIBERSORT was applied to analyze the fractions of TIICs, and each dot represents one sample. The mean±SD for each cell subtype including total macrophages (**A**), total mast cells (**B**), resting mast cells (**C**), activated mast cells (**D**), M0 macrophages (**E**), M1 macrophages (**F**), M2 macrophages (**G**) and for the M2/M1 ratio (**H**) was calculated and compared using one-way ANOVA. *P<0.05; ***P<0.001.

The fractions of total DCs, resting DCs and monocytes were lower in gastric cancer than in normal tissue (*P*<0.05 or *P*<0.001, Figure 3B, 3C, 3G). The fractions of total and resting mast cells were strongly decreased in gastric cancer compared to normal tissue(*P*<0.001, Figure 4B, 4C), whereas the activated mast cell fraction increased slightly (*P*<0.05, Figure 4D). The total macrophage fraction increased significantly in gastric cancer compared with normal tissue (*P*<0.001, Figure 4A), which contributed to the incremental increases in the M0 and M1 macrophage fractions (*P*<0.001, Figure 4E, 4F). The M2 fraction decreased in cancer tissue (*P*<0.001, Figure 4G). Correspondingly, the ratio of M2/M1 was lower in gastric cancer than that in normal tissue (Figure 4H). In addition, resting NK cells, activated NK cells, activated DCs, eosinophils and neutrophils did not differ between tumor and normal tissues.

### Immune cell composition and its prognostic significance in different stages of gastric cancer

**Figure 5:**
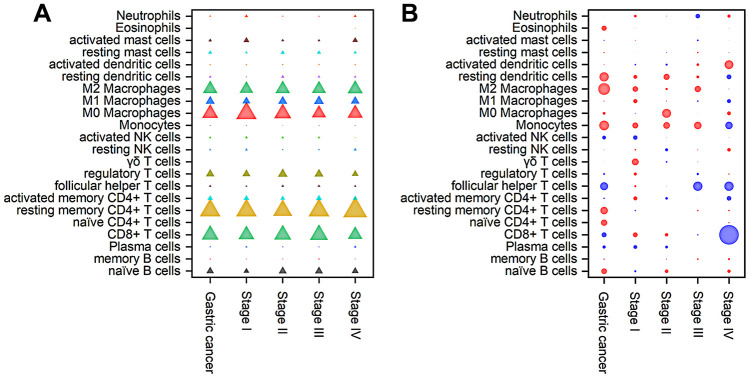
****Composition of TIICs (**A**) and a bubble heat map showing the associations between OS and immune cell subfractions (**B**) in different stages of gastric cancer. The red and blue colours in the heatmap represent negative and positive correlations, respectively, between TIICs and OS, while bubble size indicates the level of statistical significance.

The composition of TIICs in different stages of gastric cancer was analyzed and is shown in Figure 5A and Supplementary Table 2. The results illustrated that TAMs (31.94%), resting memory CD4^+^ T cells (16.49%), CD8^+^ T cells (13.45%) and Treg cells (6.81%) were abundant in gastric cancer, whereas naive CD4^+^ T cells (0.01%), eosinophils (0.43%), δγ T cells (0.34%), monocytes (0.77%) and memory B cells (1.18%) were sparse. From stage I to IV, the fraction of δγ T cells continuously increased, whereas the proportions of activated NK cells and M0 macrophages continuously declined. In addition, the fractions of Tfh cells, Treg cells, resting DCs and resting mast cells increased in stage II and then decreased as the stage advanced. The proportions of naive CD4^+^ T cells and eosinophils suddenly increased in stage IV disease.

To further investigate the prognostic value of TIICs, Cox regression analysis was applied to analyze the associations between overall survival (OS) and immune cell subfractions in different stages of gastric cancer. As shown in Figure 5B, a higher proportion of Tfh cells indicated prolonged OS (hazard ratio (HR)=0.61, 95% confidence interval (CI) 0.42~0.90, *P*<0.05), especially in stages III and IV, whereas worse OS was associated with relatively high fractions of M2 macrophages (HR=1.47, 95% CI 1.12~1.94, *P*<0.05), resting DCs (HR=1.40, 95% CI 1.06~1.84, *P*<0.05) and monocytes (HR=1.42, 95% CI 1.07~1.87, *P*<0.05). In stage I tumors, immune cells had little influence on the OS. In stage II tumors, relatively poor OS was correlated with an increased fraction of M0 macrophages (HR=2.05, 95% CI 1.12~3.78, *P*<0.05). In stage III tumors, prolonged OS was associated with a relatively high proportion of Tfh cells (HR=0.62, 95% CI 0.41~0.94, *P*<0.05). In stage IV tumors, an increased number of CD8^+^ T cells was significantly associated with prolonged OS (HR=0.20, 95% CI 0.09~0.46, *P*<0.01). Therefore, the correlation between TIICs and OS displayed great diversity among different stages.

### Identification of differentially expressed IRGs

**Figure 6:**
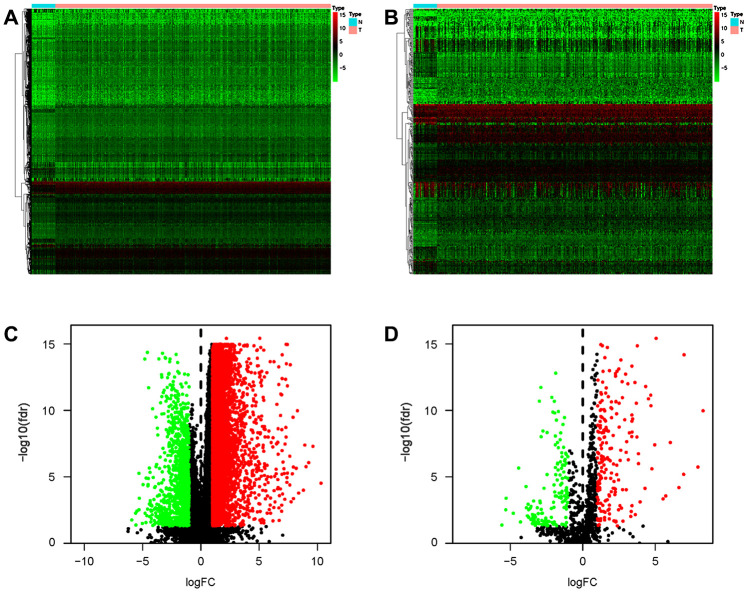
**Differentially expressed IRGs in the gastric cancer cohort.** Heatmap of DEGs (**A**) and differentially expressed IRGs (**B**). Volcano plot of DEGs (**C**) and differentially expressed IRGs (**D**). Blue and red dots represent DEGs, and black dots represent genes that were not differentially expressed.

As IRGs can reflect the immune status of cancer patients, we extracted IRGs with differential expression in gastric cancer patients from transcriptomic RNA-seq data for further analyses. First, Wilcoxon signed-rank test was applied to identify differentially expressed genes (DEGs) between gastric cancer and normal tissue. The results showed that a total of 6749 DEGs were screened, including 5601 upregulated and 1148 downregulated genes (Figure 6A, 6C). Among these DEGs, we further identified 345 differentially expressed IRGs, including 198 upregulated and 147 downregulated IRGs (Figure 6B, 6D).

### Characterization of hub IRGs

**Figure 7:**
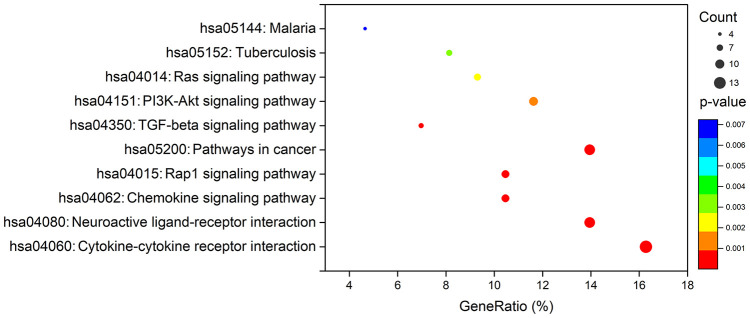
**KEGG analysis of survival-associated IRGs.**

**Figure 8:**
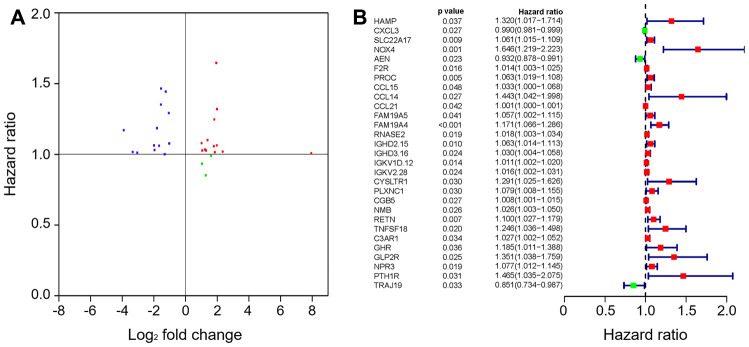
**The hub IRGs in the gastric cancer cohort.** (**A**) Identification of hub genes. (**B**) Prognostic value of hub genes.

**Figure 9:**
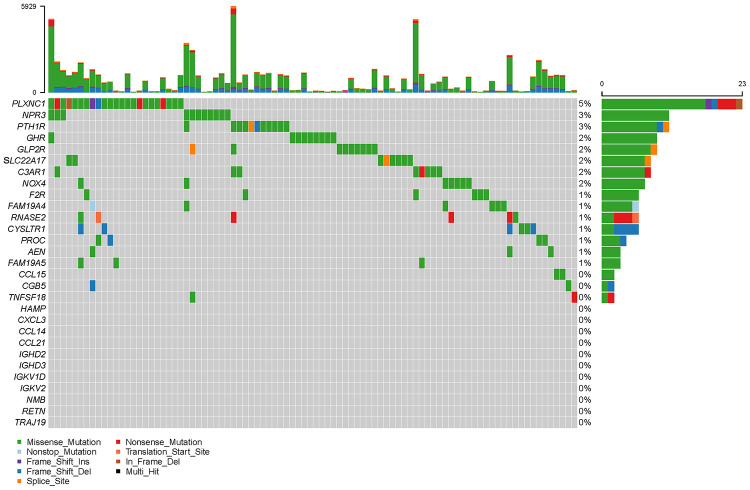
**Mutation frequencies of hub IRGs.**

To create a valuable prognostic signature, univariate Cox analysis was conducted to screen IRGs associated with the OS of gastric cancer patients. In total, 100 genes were found to be significantly associated with clinical outcomes (*P*<0.05). Then, Gene Ontology (GO) enrichment analysis of these survival-associated IRGs showed that “positive regulation of ERK1 and ERK2 cascade”, “positive regulation of cytosolic calcium ion concentration” and the “inflammatory response” were the three most significant biological process terms; the “extracellular region”, the “extracellular space” and “integral component of plasma membrane” were the three most significant cellular component terms; and “growth factor binding”, “growth factor activity”, and “peptide hormone binding” were the three most significant molecular function terms (Table 1). Cytokine-cytokine receptor interaction was found to be the most frequently enriched Kyoto Encyclopedia of Genes and Genomes (KEGG) pathway (Figure 7). Furthermore, 29 hub IRGs were ascertained to be differentially expressed in gastric cancer and closely related to the OS (Figure 8A). A forest plot of hazard ratios indicated that most of these hub IRGs were high-risk factors (Figure 8B). Owing to the potential prognostic significance of these hub IRGs, their molecular characteristics related to genomic alterations were further analyzed. The results showed that these hub IRGs were unstable in gastric cancer and missense mutations were the most commonly occurring type (Figure 9).

**Table 1:** GO term enrichment analysis of survival-associated IRGs.

**Ontology**	**ID**	**Description**	***P*. adjust**	**Count**
Biological process	GO:0070374	positive regulation of ERK1 and ERK2 cascade	4.88E-10	12
	GO:0007204	positive regulation of cytosolic calcium ion concentration	1.15E-08	10
	GO:0006954	inflammatory response	1.88E-08	14
	GO:0008284	positive regulation of cell proliferation	2.67E-08	15
	GO:0030335	positive regulation of cell migration	1.78E-07	10
	GO:0000187	activation of MAPK activity	6.37E-07	8
	GO:0006935	chemotaxis	1.55E-06	8
	GO:0010595	positive regulation of endothelial cell migration	2.24E-06	6
	GO:0060326	cell chemotaxis	1.26E-05	6
	GO:0007200	phospholipase C-activating G-protein coupled receptor signaling pathway	1.36E-05	6
Cellular component	GO:0005576	extracellular region	3.20E-13	32
	GO:0005615	extracellular space	4.77E-11	27
	GO:0005887	integral component of plasma membrane	1.10E-07	23
	GO:0009986	cell surface	6.28E-07	14
	GO:0005886	plasma membrane	2.12E-05	36
	GO:0005623	cell	7.81E-05	6
	GO:0002116	semaphorin receptor complex	1.02E-03	3
	GO:0045121	membrane raft	2.13E-03	6
	GO:0043235	receptor complex	2.36E-03	5
	GO:0005768	endosome	1.72E-02	5
Molecular function	GO:0019838	growth factor binding	1.38E-07	6
	GO:0008083	growth factor activity	8.02E-07	9
	GO:0017046	peptide hormone binding	6.83E-06	5
	GO:0050431	transforming growth factor beta binding	5.09E-05	4
	GO:0008009	chemokine activity	7.63E-05	5
	GO:0019955	cytokine binding	8.72E-05	4
	GO:0005125	cytokine activity	1.63E-04	7
	GO:0004888	transmembrane signaling receptor activity	4.65E-04	7
	GO:0008201	heparin binding	8.69E-04	6
	GO:0005102	receptor binding	1.22E-03	8

### Prognostic signature for gastric cancer patients

**Figure 10:**
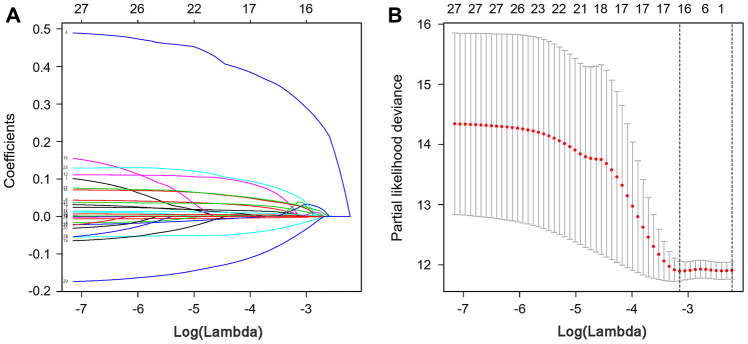
**LASSO coefficient profiles of hub IRGs.** The coefficient profiles (**A**) and partial likelihood deviance (**B**) of hub IRGs.

**Figure 11:**
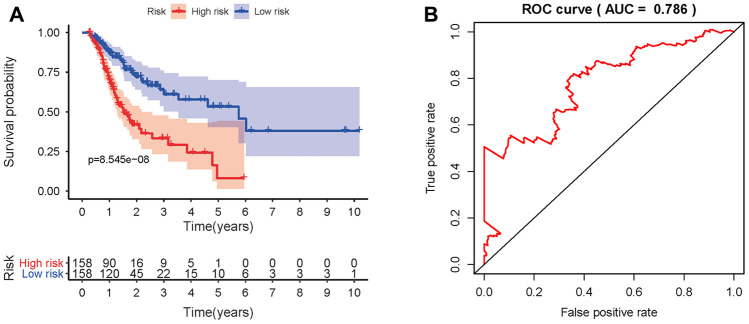
**Prognostic value of the prognostic model.** (**A**) Kaplan-Meier plot depicting the survival probabilities predicted by the prognostic model over time for the high- (red) and low-risk (blue) groups. (**B**) Survival-dependent ROC analysis of the prognostic value of the prognostic model.

**Figure 12:**
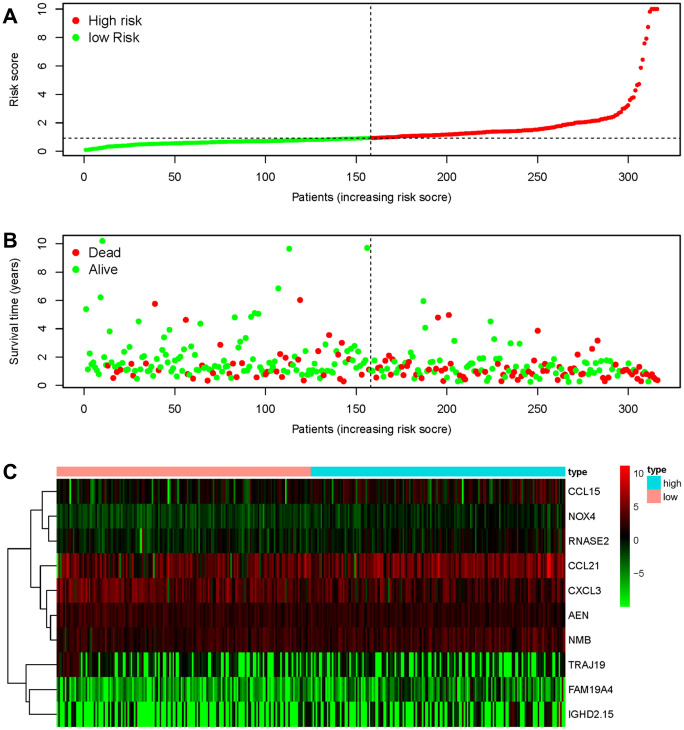
**Discriminatory capability of the IRG-based prognostic signature.** (**A**) Rank of the prognostic signature and distribution of the high- and low-risk groups. (**B**) Survival status of patients in the high- and low-risk groups distinguished by dotted lines. (**C**) Heatmap of IRGs used to construct the prognostic signature.

To develop a prognostic indicator for the prediction of survival outcomes, LASSO Cox regression analysis was carried out, and ten hub IRGs were screened to construct a prognostic signature (Figure 10). Kaplan-Meier plots indicated that the prognostic signature could predict the survival probability of gastric cancer patients (Figure 11A). The area under the receiver operating characteristic (ROC) curve was 0.786, indicating the moderate potential for survival prediction (Figure 11B). Further validation illustrated that the constructed prognostic model could separate the survival status of gastric cancer patients into high- and low-risk groups (Figure 12). The formula was as follows: [Expression level of *CXCL3* * (-0.0067)] + [Expression level of *NOX4* * 0.5146] + [Expression level of *AEN* * (-0.0610)] + [Expression level of *CCL15* * 0.0420] + [Expression level of *CCL21* * 0.0012] + [Expression level of *FAM19A4* * 0.1248] + [Expression level of *RNASE2* * 0.0183] + [Expression level of *IGHD2.15* * 0.0956] + [Expression level of *NMB* * 0.0432] + [Expression level of *TRAJ19* * (-0.1860)].

### Confirmation of the prognostic signature

**Figure 13:**
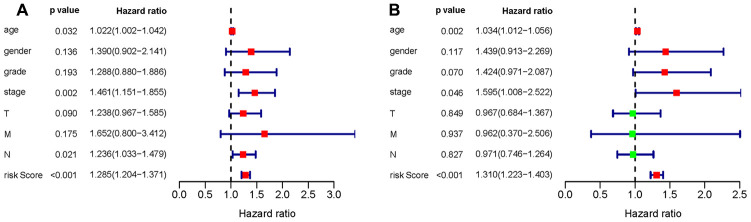
****Univariate (**A**) and multivariate (**B**) Cox regression analyses of the gastric cancer cohort.

To verify whether the constructed prognostic signature could function as an independent predictor, univariate and multivariate Cox regression analyses were carried out and compared. The results showed that the prognostic signature was an independent predictor of the prognosis of gastric cancer patients after other parameters were adjusted, including age, sex, tumor grade and TNM stage (Figure 13).

### Validation of the associations of IRGs with TIICs

**Figure 14:**
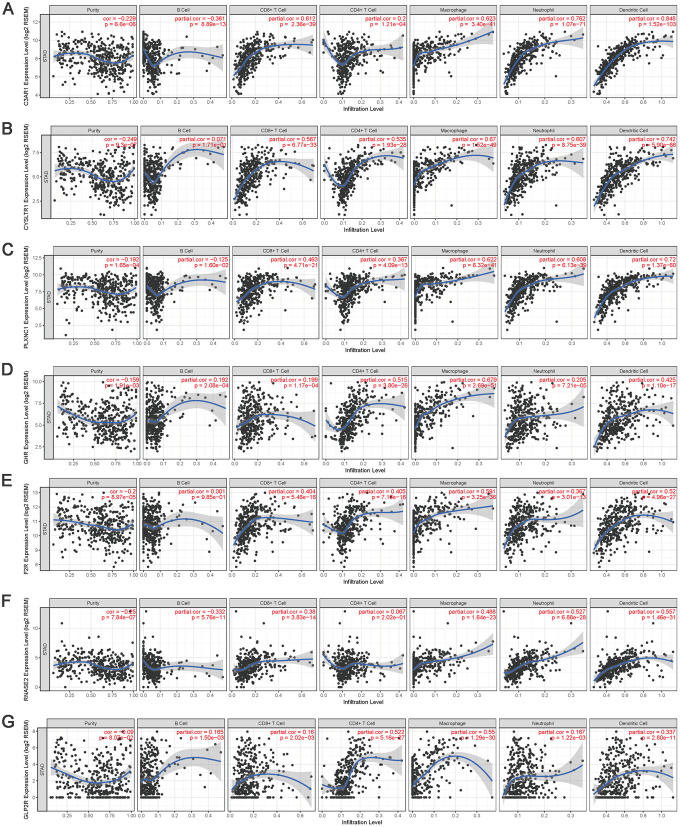
**The correlation between the hub IRGs and TIICs.** The expression levels of C3AR1 (**A**) CYSLTR1 (**B**) PLXNC1 (**C**) GHR (**D**) F2R (**E**) RNASE2 (**F**) and GLP2R (**G**) and their associations with the infiltration levels of immune cells.

To validate the relationships between IRGs and TIICs, TIMER was used to visualize the correlations between the expression of hub IRGs and the infiltrating levels of B cells, CD8^+^ T cells, CD4^+^ T cells, macrophages, neutrophils and DCs in the TME. The results showed that most of the hub IRGs were significantly associated with the abundances of TIICs, especially *C3AR1*, *CYSLTR1*, *PLXNC1*, *GHR*, *F2R*, *RNASE2* and *GLP2R*, which are shown in Figure 14.

## DISCUSSION

Cancer is a genetic and immune-mediated disease, and tumor fate is shaped by the host immune system through the activation of innate and adaptive immune mechanisms, named cancer immunoediting [22]. In cancer patients, the balance between tolerance and immunity is influenced by a complex set of tumor, host and environmental factors [23]. Therefore, many efforts have been devoted to enhancing antitumor immunity by exploring immune cell-based vaccines, targeting immune checkpoints, or improving the immune microenvironment. Although immunotherapy is proven to be an effective therapeutic approach in a variety of cancers, only a subset of patients exhibit durable responses [23]. Gastric cancer has strong heterogeneity, and the treatment outcomes and prognosis are affected by tumor location, subtype, age, sex, etc. How to govern the strength and timing of antitumor responses are key problems that deserve careful consideration.

Previous studies have proven that TIICs are highly relevant to tumorigenesis, invasion, and metastasis. The interactions between TIICs and tumor cells are considered to be directly associated with the physical destruction of the tumor cells, tumor burden reduction, and clinical prognosis improvement. An increasing number of studies, however, have suggested that tumor progression may be promoted by aberrant infiltration of immune cells [11]. Therefore, immune cells may play a dual role in stimulating antitumor immunity or promoting tumor development in cancer patients. In this study, the composition and prognostic value of TIICs in gastric cancer patients were analyzed based on the deconvolution of bulk gene expression data from a large set of samples. We found that there were considerable differences in TIIC compositions and that relatively high fractions of M2 macrophages, resting DCs and monocytes indicated relatively poor OS for patients with gastric cancer.

Macrophages are highly plastic cells that can be divided into classical M1 and alternative M2 phenotypes based on their function [7, 24]. It is generally accepted that M1 macrophages participate in inflammatory reactions and T lymphocyte-medicated antitumor immunity, whereas M2 macrophages have pro-tumorigenic properties [24]. Tumor associated macrophages (TAMs) are one type of main immune cells and mostly have an M2 phenotype. In gastric cancer, however, we observed increases in the M0 and M1 macrophage fractions and decrease in the M2 macrophage fraction, leading to incremental increase in the M1/M2 ratio, which seems to be a good tendency. Thorsson et al. considered that a relatively high M1/M2 ratio might reiterate the local proinflammatory state in patients with this phenotype [24]. Li et al. found that an acidic polysaccharide could reprogram TAMs into an M1 phenotype to restore local immune surveillance in the TME [25]. However, an increased number of M0 macrophages predicted relatively poor OS in stage II tumors, indicating that circulating macrophages can be recruited into tumors to alter the TME and promote tumor progression. Therefore, activation of macrophages with different properties in various microenvironments may reverse their function and our results further suggested that TAMs could be used as diagnostic and prognostic biomarkers in gastric cancer.

Probst et al. revealed that resting DCs could induce peripheral CD8^+^ T cell tolerance through PD-1 and CTLA-4 molecules, whereas activated DCs could efficiently prime naive, endogenous cytotoxic T lymphocyte (CTL) to expand and to develop effector functions [26, 27]. However, the immune microenvironment can promote selective development of regulatory DC subsets [28–30], and sometimes activated DCs stimulate the proliferation of Tregs [31, 32]. Thus, the functions of DCs are discrepancies in different subsets and may be affected by tumor-induced immunosuppression microenvironment [28–30, 33, 34]. In addition, activated NK cells, eosinophils and neutrophils are important for antitumor immunity, and their accumulation and infiltration in tumor and peritumoral tissues are closely associated with prognosis. In this study, the proportion of eosinophils suddenly increased in stage IV tumors, but there was no significant difference between eosinophils and OS. A growing number of observations revealed that eosinophils could make a great difference to tumor initiation and progression, but they could also display regulatory functions towards other immune cells or direct cytotoxic functions against tumor cells depending on the milieu [35–37]. Thus, it is worthy of eosinophil research to understand how they operate in the TME, which will hopefully unearth new clues for cancer immunotherapy.

Furthermore, we found that relatively high proportions of Tfh cells and CD8^+^ T cells strongly predicted prolonged OS in advanced gastric cancer. The fractions of plasma cells and resting memory CD4^+^ T cells decreased, while those of activated memory CD4^+^ T cells and Tregs increased in gastric cancer. The proportion of naive CD4^+^ T cells increased in stage IV tumors, but there was no significant influence on the OS of gastric cancer patients. Previous studies indicated that the abundance of naive CD4^+^ T cells is often correlated with poor prognosis of cancer patients [38, 39]. Our observation might suggest that the function of DCs was impaired to activate naive CD4^+^ T cells in the advanced patients. Thus, it may be difficult to stimulate the T cell-mediated antitumor immune response in patients with advanced-stage gastric cancer, which may be related to the poor prognosis and high mortality of advanced patients.

The TME is correlated with the proliferation, invasion, metastasis and immune escape of tumor cells, in which tumor cells can induce immunosuppression by mimicking immune cells through IRG expression [33, 40]. The TCGA database provides abundant information on DEGs in various cancers and survival outcomes. Recent studies integrated the expression profiles of survival-associated IRGs with clinical information to develop individualized prognostic signatures for cancer patients and elucidated that the relationships between immune-based signatures and immune cell infiltration could reflect the status of the immune microenvironment [15, 16, 41–45]. Thus, the investigation of IRGs is particularly critical to provide more prognostic information and predict responses to therapy. By applying Wilcoxon signed-rank test and univariate Cox analysis, 29 hub IRGs that were differentially expressed in gastric cancer and significantly associated with the OS were identified. Then, LASSO Cox regression analysis was conducted and ten hub IRGs were ascertained to construct the formula for prognostic model. The AUC of the ROC curve reached 0.786. The correlations of OS with age, sex, tumor grade, TNM stage and the risk score were analyzed and demonstrated the favorable clinical viability of the constructed model. Thus, an independent predictor was successfully modelled for outcome prediction, which could provide practical guidance to adjust treatment strategies and improve the antitumor immune responses of gastric cancer patients.

In summary, the present study includes several *in silico* analyses on the gene expression profiles of 374 unrelated tumor samples from gastric cancer patients with known clinical follow-up data. First, CIBERSORT was applied to estimate the relative proportions of 22 types of immune cells in these tumor samples. Both innate and adaptive immune cells were changed to various degrees in gastric cancer samples compared to normal tissue samples and among different tumor stages. Second, prognostic analysis showed that relatively poor OS was associated with relatively high fractions of M2 macrophages, resting DCs and monocytes, whereas an increased number of CD8^+^ T cells was significantly associated with prolonged OS. Third, we calculated the prognostic value of IRGs and built an independent predictor for gastric cancer patient outcome prediction. Ultimately, we substantiated the significant correlation between hub IRGs and TIICs and further confirmed the research significance of our analyses. These results may be helpful for improving immunotherapeutic regimens or enhancing antitumor immunity in gastric cancer patients.

## MATERIALS AND METHODS

### Data acquisition

Transcriptomic RNA-seq data for gastric cancer samples were downloaded from the TCGA database, including data for 374 primary gastric cancer and 32 normal tissues. Mutation data and clinicopathological information were also collected, including age, sex, tumor grade, TNM stage and OS. The primary tumor characteristics and clinical information are showed in Supplementary Table 1. A list of IRGs was derived through the Immunology Database and Analysis Portal (ImmPort) database (https://www.immport.org/) [46].

### Composition analyses of immune cells

CIBERSORT, a deconvolution algorithm [5, 12], was applied to estimate the relative proportions of 22 types of TIICs in gastric cancer using normalized gene expression data. These TIICs included resting memory CD4^+^ T cells, activated memory CD4^+^ T cells, Tfh cells, Tregs, γδ T cells, CD8^+^ T cells, naive CD4^+^ T cells, naive B cells, memory B cells, plasma cells, resting NK cells, activated NK cells, macrophages (M0, M1 and M2), resting DCs, activated DCs, resting mast cells, activated mast cells, eosinophils, neutrophils and monocytes. The immune cell profiles for each sample and the mean values for gastric cancer and normal tissue were calculated. A set of reference gene expression values (a “signature matrix” of 547 genes) considered a minimal representation for each cell type was used to infer cell type proportions in data from a bulk tumor sample with mixed cell types using support vector regression. The algorithm was performed using the LM22 signature matrix with 1000 permutations. *P* values were calculated by a one-way ANOVA to compare gastric cancer and normal tissue.

For evaluation of the different stages of gastric cancer, the compositions of the 22 types of TIICs were compared after each dataset was processed by a weighted average method. At the same time, Cox regression analysis was performed to judge the prognostic value of TIICs. The package language R (v3.3.2) and Bioconductor (https://www.bioconductor.org/) were used for statistical analyses. The HR and 95% CI were determined, and *P*<0.05 was considered statistically significant.

### Analysis of DEGs

The Wilcoxon signed-rank test was used to screen DEGs between gastric cancer and normal tissue based on the RNA-seq data. The false discovery rate (FDR)<0.05 and log_2_|fold change|>1 were set as the thresholds to define DEGs. Then, the identified DEGs were used to screen differentially expressed IRGs. Univariate Cox analysis was performed to estimate the associations between IRGs and the OS of gastric cancer patients. The HR was determined, and *P*<0.05 was considered significant. Then, GO and KEGG enrichment analyses were conducted to analyze the functions and potential molecular mechanisms of the screened IRGs. The intersection between differentially expressed IRGs and survival-associated IRGs was used to define hub IRGs. In addition, the genetic alterations in these hub genes were analyzed through cBioPortal (http://www.cbioportal.org/) [47, 48].

### Construction of a prognostic signature

The identified survival-associated IRGs were selected for multivariate LASSO Cox analysis to develop a prognostic signature. Kaplan-Meier analysis was used to plot the survival probability, and ROC analysis was performed to assess the validity of the prognostic signature. Gastric cancer patients were divided into high- and low-risk groups, and the prognostic value of the prognostic signature was assessed in the patients. Finally, univariate and multivariate Cox regression analyses of age, sex, tumor grade, TNM stage and the risk score were performed to verify whether the constructed prognostic signature was an independent predictor. TIMER was used to validate and visualize the relationships of hub IRGs and TIICs, including B cells, CD4^+^ T cells, CD8^+^ T cells, neutrophils, macrophages and DCs. TIMER is a web resource that incorporates 10,009 samples across 23 cancer types from the TCGA database to evaluate the clinical impacts of different TIICs on diverse cancer types. The Gene analysis in TIMER can be conducted to analyze the correlation between a given immune cell type and the expression of a selected gene [49, 50].

## Supplementary Material

Supplementary Table 1

Supplementary Table 2
