# Supplementary material for: Prognostic landscape of tumor-infiltrating immune cells and immune-related genes in the tumor microenvironment of gastric cancer
Source: Aging (Albany NY). 2020 Sep 23;12(18):17958–75. doi: 10.18632/aging.103519 (PMC7585095; doi:10.18632/aging.103519)
Supplement: Supplementary Table 1 [file aging-12-103519-s001..docx]

**Supplementary Table 1.** Primary tumor characteristics and clinical information.

| **Variable** | **Number of samples** | **Ratio (%)** | **Valid (%)** |
| --- | --- | --- | --- |
| Age at diagnosis, y |  |  |  |
| ≤50 | 29 | 7.75 | 7.86 |
| ＞50 | 340 | 90.91 | 92.14 |
| Missing | 5 | 1.34 |  |
| Tumor type |  |  |  |
| Signet ring type | 11 | 2.94 | 6.63 |
| Diffuse type | 63 | 16.85 | 37.95 |
| Tubular type | 68 | 18.18 | 40.96 |
| Mucinous type | 19 | 5.08 | 11.45 |
| Papillary type | 5 | 1.34 | 3.01 |
| Not otherwise specified (NOS) | 208 | 55.61 |  |
| Tumor grade |  |  |  |
| 1 | 10 | 2.67 | 2.75 |
| 2 | 136 | 36.37 | 37.36 |
| 3 | 218 | 58.29 | 59.89 |
| Missing | 10 | 2.67 |  |
| Gender |  |  |  |
| Male | 240 | 64.17 | 64.34 |
| Female | 133 | 35.56 | 35.66 |
| Missing | 1 | 0.27 |  |
| Race |  |  |  |
| White | 237 | 63.37 | 73.37 |
| Asian | 74 | 19.79 | 22.91 |
| African American | 11 | 2.94 | 3.41 |
| Native Hawaiian or other pacific islander | 1 | 0.27 | 0.31 |
| Missing | 51 | 13.63 |  |
| T-stage |  |  |  |
| T1 | 19 | 5.08 | 5.21 |
| T2 | 79 | 21.12 | 21.64 |
| T3 | 167 | 44.65 | 45.75 |
| T4 | 100 | 26.74 | 27.40 |
| Missing | 9 | 2.41 |  |
| N-stage |  |  |  |
| N0 | 111 | 29.68 | 31.26 |
| N1 | 96 | 25.66 | 27.04 |
| N2 | 74 | 19.79 | 20.85 |
| N3 | 74 | 19.79 | 20.85 |
| Missing | 19 | 5.08 |  |
| M-stage |  |  |  |
| M0 | 328 | 87.70 | 92.92 |
| M1 | 25 | 6.69 | 7.08 |
| Missing | 21 | 5.61 |  |
| Stage |  |  |  |
| Ⅰ | 53 | 14.17 | 15.14 |
| Ⅱ | 109 | 29.14 | 31.14 |
| Ⅲ | 150 | 40.11 | 42.86 |
| Ⅳ | 38 | 10.16 | 10.86 |
| Missing | 24 | 6.42 |  |
| Antireflux treatment |  |  |  |
| Yes | 37 | 9.89 | 20.79 |
| No | 141 | 37.70 | 79.21 |
| Missing | 196 | 52.41 |  |
| Family history of stomach cancer |  |  |  |
| Yes | 15 | 4.01 | 5.24 |
| No | 271 | 72.46 | 94.76 |
| Missing | 88 | 23.53 |  |
| Radiation treatment adjuvant |  |  |  |
| Yes | 44 | 11.76 | 23.28 |
| No | 145 | 38.77 | 76.72 |
| Missing | 185 | 49.47 |  |
| Targeted molecular therapy |  |  |  |
| Yes | 90 | 24.06 | 47.12 |
| No | 101 | 27.01 | 52.88 |
| Missing | 183 | 48.93 |  |
