# Supplementary material for: Prognostic landscape of tumor-infiltrating immune cells and immune-related genes in the tumor microenvironment of gastric cancer
Source: Aging (Albany NY). 2020 Sep 23;12(18):17958–75. doi: 10.18632/aging.103519 (PMC7585095; doi:10.18632/aging.103519)
Supplement: Supplementary Table 2 [file aging-12-103519-s002..pdf]

SUPPLEMENTARY TABLE

Supplementary Table 2. Composition of TIICs in different stages of gastric cancer.

TIICs	Gastric cancer	Stage I	Stage II	Stage III	Stage IV
Naive B cells	0.060435	0.04518	0.064665	0.06292	0.056648
Memory B cells	0.011808	0.00851	0.010541	0.013629	0.008848
Plasma cells	0.013114	0.01596	0.012274	0.011375	0.019927
$CD8^+$ T cells	0.134547	0.126714	0.137343	0.143324	0.115744
Naive $CD4^+$ T cells	0.0001	0	0	0	0.000989
Resting memory $CD4^+$ T cells	0.164883	0.163031	0.151987	0.167391	0.190702
activated memory $CD4^+$ T cells	0.043826	0.045551	0.038578	0.04878	0.038844
T follicular helper cells	0.02115	0.016063	0.023463	0.023315	0.019258
Treg cells	0.068071	0.065053	0.076524	0.070416	0.054654
Gamma delta T cells	0.003393	0.001758	0.002024	0.003686	0.003976
Resting NK cells	0.017307	0.023442	0.014708	0.01532	0.025292
Activated NK cells	0.019013	0.023218	0.020494	0.019989	0.007192
Monocytes	0.007745	0.007451	0.006625	0.007135	0.007901
M0 macrophages	0.128218	0.163447	0.137092	0.115204	0.122443
M1 macrophages	0.073627	0.063678	0.072022	0.079088	0.070016
M2 macrophages	0.117545	0.108058	0.116887	0.113737	0.122498
Resting dendritic cells	0.021736	0.010988	0.027527	0.022215	0.019511
Activated dendritic cells	0.013182	0.013092	0.014284	0.013338	0.013465
Resting mast cells	0.031781	0.021085	0.034657	0.033436	0.025814
Activated mast cells	0.028294	0.047219	0.023785	0.021637	0.044962
Eosinophils	0.004262	0.003541	0.002269	0.00221	0.00769
Neutrophils	0.015961	0.026962	0.012249	0.011856	0.023628
